# Androgen-induced masculinization in rainbow trout results in a marked dysregulation of early gonadal gene expression profiles

**DOI:** 10.1186/1471-2164-8-357

**Published:** 2007-10-04

**Authors:** Daniel Baron, Jérôme Montfort, Rémi Houlgatte, Alexis Fostier, Yann Guiguen

**Affiliations:** 1 INRA, UR1037 SCRIBE, IFR140, Ouest-Genopole, F-35000 Rennes, France; 2 Institut National de la Santé et de la Recherche Médicale, L'institut du thorax, INSERM U533, Faculté de Médecine, 1 rue Gaston Veil, BP 53508, 44035 Nantes cedex 1, France

## Abstract

**Background:**

Fish gonadal sex differentiation is affected by sex steroids treatments providing an efficient strategy to control the sexual phenotype of fish for aquaculture purposes. However, the biological effects of such treatments are poorly understood. The aim of this study was to identify the main effects of an androgen masculinizing treatment (11β-hydroxyandrostenedione, 11βOHΔ4, 10 mg/kg of food for 3 months) on gonadal gene expression profiles of an all-female genetic population of trout. To characterize the most important molecular features of this process, we used a large scale gene expression profiling approach using rainbow trout DNA microarrays combined with a detailed gene ontology (GO) analysis.

**Results:**

2,474 genes were characterized as up-regulated or down-regulated in trout female gonads masculinized by androgen in comparison with control male or female gonads from untreated all-male and all-female genetic populations. These genes were classified in 13 k-means clusters of temporally correlated expression profiles. Gene ontology (GO) data mining revealed that androgen treatment triggers a marked down-regulation of genes potentially involved in early oogenesis processes (GO 'mitotic cell cycle', 'nucleolus'), an up-regulation of the translation machinery (GO 'ribosome') along with a down-regulation of proteolysis (GO 'proteolysis', 'peptidase' and 'metallopeptidase activity'). Genes considered as muscle fibres markers (GO 'muscle contraction') and genes annotated as structural constituents of the extracellular matrix (GO 'extracellular matrix') or related to meiosis (GO 'chromosome' and 'meiosis') were found significantly enriched in the two clusters of genes specifically up-regulated in androgen-treated female gonads. GO annotations 'Sex differentiation' and 'steroid biosynthesis' were enriched in a cluster of genes with high expression levels only in control males. Interestingly none of these genes were stimulated by the masculinizing androgen treatment.

**Conclusion:**

This study provides evidence that androgen masculinization results in a marked dysregulation of early gene expression profiles when compared to natural testicular or ovarian differentiation. Based on these results we suggest that, in our experimental conditions, androgen masculinization proceeds mainly through an early inhibition of female development.

## Background

The embryonic gonad has the potential to develop into a fully functional organ able to produce the gametes necessary for sexual reproduction. Sex differentiation is a crucial step in this developmental process and is considered as the differentiation from a bipotential gonadal primordium towards a testis or an ovary. In teleostean fish, sex differentiation can be controlled by *in vivo *treatments with sex steroids (reviewed in [1]) as in reptiles and amphibians and to some extent in birds (reviewed in [2-4]). In fish, these steroid treatments are often able to induce fully functional sex-inversed phenotypes and these treatments have been widely used to produce all-male or all-female populations of fish for aquaculture purposes [5]. Many studies have been focused on the role of these hormones during gonadal sex differentiation highlighting for instance the crucial role of estrogens in ovarian differentiation [1]. However, most of the studies performed thus far were focused on a very small number of well characterized genes, proteins or hormones and mostly on natural gonadal differentiation.

Rainbow trout, *Oncorhynchus mykiss*, has a male heterogametic XY genetic system and we experimentally produced XX and YY males allowing the production of genetically all-male and all-female populations [6]. These all-male or all-female populations provide a unique opportunity to work on numerous animals for which the normal gonadal development as testis or ovary is known *a priori*. Using the extensive collection of expressed sequenced tags (ESTs) obtained through sequencing projects in trout as a resource [7,8], we designed and built a DNA microarray in order to characterize, on a genome-wide scale, the mechanisms by which 11β-hydroxyandrostenedione (11βOHΔ4), a natural androgen in fish [9,10] is able to masculinize the embryonic ovary.

Using this genome-wide approach we characterized 2,474 genes (2372 microarray and 102 real-time RT-PCR gene expression profiles) with a clear differential temporal expression profile in females masculinized by androgen. We classified these genes in 13 different clusters of correlated temporal expression profiles, and searched within these clusters for significant enrichment in Gene Ontology (GO) terms. This strategy allowed us to define a few very clear biological trends potentially explaining how androgen induces masculinization of female fish. Our results clearly demonstrate that masculinization with androgen proceeds through a marked dysregulation of gene expression profiles, including a quick down-regulation of the ovarian pathway. Surprisingly, most of the genes over-expressed during natural testicular differentiation were not restored by the androgen-induced masculinization suggesting that the inhibition of female gonadal development is the main required step sufficient for building a testis.

## Results

The complete dataset is available through the National Center for Biotechnology Information (NCBI), in the Gene Expression Omnibus database [11] under the GSE7018 accession number. After statistical filtering, 2,474 expression profiles (2372 microarray and 102 real-time RT-PCR gene expression profiles, data available as supplemental material in Additional file 1) were identified as being characteristic for either natural differentiation (ovarian or testicular differentiation) or androgen-induced masculinization (trans-differentiating gonads). Among these 2,474 expression profiles, 73% (1,805) were associated with genes with significant homologies with well characterized proteins in Swissprot or Prodom databases (the complete list of clones and their annotations is available as supplemental material in Additional file 2).

### Biological sample clustering and histology

**Figure 1:**
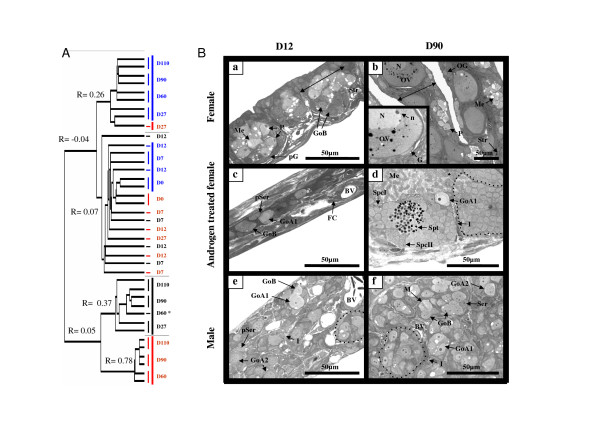
**Classification of gonad samples and histological analysis of some characteristic gonadal stages**. (A) Dendrogram of the samples ranked using a hierarchical clustering. Gonad samples are colorized according to the sex i.e., red for females, blue for males and black for androgen-treated females. Correlation coefficients (R) of the last branches of a cluster are given. (B) Histology of the gonads from the female control group (a, b), the androgen-treated group (c, d), and the male control group (e, f) at 12 days (D12) and 90 days (D90) after the beginning of the androgen treatment. BV: blood vessel; FC: fibroblast like cell; Go (A1/A2/B): gonia type (A1/A2/B); I: Interstitial space; M: mitosis; Me: meiosis; N: nucleus; n: nucleolus; OV: ovocyte; P: pachytene stage of meiosis; (p)G: (pre)granulosa cell ; (p)S: (pre)sertoli cell ; Spc(I/II): spermatocyte I/II; Spt: spermatid; Str: ovarian stroma.

This analysis was carried out on fish sampled at several stages of development from the onset of the free swimming period (Day 0 = D0), when fish first started to be fed with the androgen treatment until 110 days after the beginning of the treatment (D110). Unsupervised hierarchical clustering of samples (Fig 1A) reveals 3 main groups of correlated samples according to their global gene expression profiles i.e., late ovarian samples (D60 to D110, correlation coefficient R = 0.78), middle and late gonad samples of androgen-treated fish gonads (D27 to D110, R = 0.37) and middle and late testicular samples (D27 to D110, R = 0.26). All early samples (D0 to D12) cluster together with a weak correlation (R = 0.07), indicating that differences in the expression profiles of these early samples are rather large. Histological analysis of gonads at D12 (Fig. 1B, panels a, c, e) reveals characteristic features of differentiating gonads in control fish with the first appearance of ovarian meiosis and lamellar structures in females, or spermatogonia cysts in males. At this time-point (D12), gonads of androgen-treated females appear as a thin structure characterized by scattered germ cells in a predominant stroma of conjunctive tissue with fibroblast like cells. After 90 days of treatment (Fig. 1B, panels b, d, f) the gonads of androgen-treated females display a classical testicular organization with cysts of germ cells engaged at various stages of meioses whereas the control male gonads show the same organization but with only gonial mitosis. At D90, the control female gonads contain previtellogenic oocytes surrounded by flattened granulosa cells and oogonia within clearly organized ovarian lamellae.

### Global analysis of gene expression profiles

**Figure 2:**
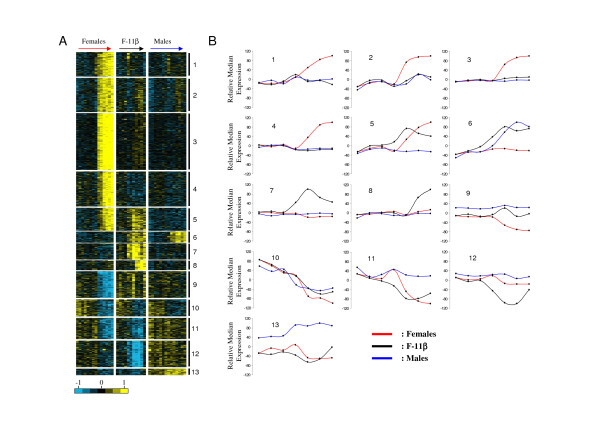
**Gene expression profiles following androgen treatment in female rainbow trout**. (A) Heat map of the 2,474 differentially expressed genes in rainbow trout gonads following androgen treatment. The 40 biological samples are ordered according to the experimental groups (red for females; blue for males; black for androgen-treated females) and to the developmental stage (D0 to D110 from left to right). (B) Relative median gene profile of the 13 clusters of temporally correlated gene expression profiles.

The 2,474 expression profiles were analyzed using a k-means clustering (with k = 13) in order to individualize clusters of genes with similar expression profiles (Fig. 2). These expression profiles and the 13 k-means clusters are available online as a browseable file [12]. Among these clusters, clusters 1 to 4 are characterized by a specific high expression levels of a very large number of genes (N = 1,204) specific to the late ovarian samples (D60 to D110). These clusters could have been merged as their expression profiles seem very similar. In cluster 5 (N = 184) these high expression levels in the female group after D60 are also present in the androgen-treated group after D27. Cluster 6 (N = 89) contains genes with increasing expression profiles starting from D16 in androgen-treated females and from D27 in control males. Clusters 7 (N = 132) and 8 (N = 77) are characterized by an early (starting from D27, cluster 7) or late (starting from D90, cluster 8) increase in gene expression specific to the androgen-treated females. Genes in cluster 9 (N = 214) display a late down-regulation starting from D60 in control female gonads. Cluster 10 (N = 133) is the only cluster that does not show any difference between males, females and androgen-treated females and contains genes with continuously decreasing expression levels from D0 to D110. Cluster 11 (N = 172) contains genes down-regulated both in females (from D60) and androgen-treated females (from D27). Cluster 12 (N = 214) is characterized by gene expression levels specifically down-regulated (from D27) in androgen-treated females. However, this down-regulation is not maintained after the end of the treatment (i.e. after D90). Cluster 13 is the smallest cluster in terms of the number of genes (N = 55) and it displays high expression levels specifically in males throughout all the sampling times (from D0 to D110). Expression levels in androgen-treated females slightly increase after completion of the treatment (> D90).

### Annotation of gene clusters using Gene Ontology (GO)

We then searched for Gene ontology (GO) terms significantly enriched in these groups of correlated expression profiles compared to the overall GO terms found. Among the 1,805 genes with an annotation we selected 1,276 unique genes annotated with an official name, and 1,133 were found to be associated with at least one GO category. The top 5 significantly enriched GO terms for each of the clusters are described in Table 1 and 2 and the complete analysis is available online (as supplemental material in Additional file 3).

**Table 1:** The top 5 most significant Gene Ontology (GO) terms significantly enriched in K-means clusters 1 to 5

**KM**	**Term (GO ID)**	**P-Value**	**RE**	**(n/N)**
1 to 5	regulation of progression through cell cycle (74)	0.0002	1.36	(50/62)
	**nucleolus **(5730)	0.0012	1.49	(23/26)
	mitotic cell cycle (278)	0.0016	1.37	(35/43)
	macromolecule biosynthesis (9059)	0.0029	1.22	(74/102)
	protein biosynthesis (6412)	0.0031	1.23	(68/93)
1	cytoplasmic membrane-bound vesicle (16023)	0.0063	3.00	(7/25)
	telomere maintenance (723)	0.0071	6.40	(3/5)
	ion homeostasis (50801)	0.0095	3.50	(5/15)
	gastrulation (7369)	0.0147	3.90	(4/11)
	digestion (7586)	0.0216	4.50	(3/7)
2	cellular respiration (45333)	0.0000	5.90	(7/8)
	translation regulator activity (45182)	0.0000	3.20	(13/27)
	aerobic respiration (9060)	0.0001	6.70	(5/5)
	pyrophosphatase activity (16462)	0.0004	2.10	(21/68)
	GTPase activity (3924)	0.0012	2.80	(10/24)
3	**mitosis **(7067)	0.0040	2.10	(12/26)
	rRNA binding (19843)	0.0064	3.30	(5/7)
	nuclear hormone receptor binding (35257)	0.0088	3.70	(4/5)
	replication fork (5657)	0.0141	2.90	(5/8)
	interphase of mitotic cell cycle (51329)	0.0154	2.10	(9/20)
4	hydrogen-transporting ATPase activity (46961)	0.0009	4.20	(6/10)
	ATP biosynthesis (6754)	0.0018	3.90	(6/11)
	cell growth (16049)	0.0053	3.30	(6/13)
	transcription factor binding (8134)	0.0094	2.20	(10/32)
	chromatin assembly (31497)	0.0169	3.50	(4/8)
5	**ribosome **(5840)	0.0000	3.40	(18/48)
	ribonucleoprotein complex (30529)	0.0000	2.70	(26/88)
	cytoplasm organization and biogenesis (7028)	0.0003	2.90	(12/37)
	cytosolic large ribosomal subunit (5842)	0.0005	4.90	(6/11)
	ribosome biogenesis and assembly (42254)	0.0006	2.90	(11/34)

**Figure 3:**
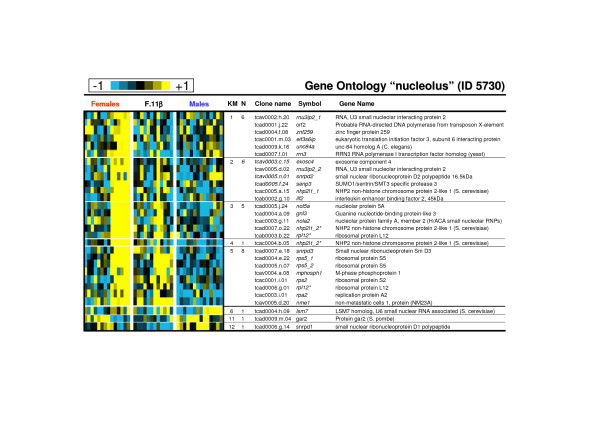
**Expression profiles of genes belonging to the Gene Ontology term "nucleolus" (ID 5730)**. Clones with similar gene names that belong to the same contig (ensemble of clones with overlapping sequences) but displayed very different expression profiles were excluded from the table (when the expression profiles were not too different the gene symbol are marked with an asterisk). Clones with similar gene names and belonging to different contigs were annotated as gene symbol_1 and gene symbol_2 as they could be considered as potentially duplicated genes or differential splicing forms. KM: K-means cluster number. N: number of genes belonging to the GO term in the specified cluster.

**Figure 4:**
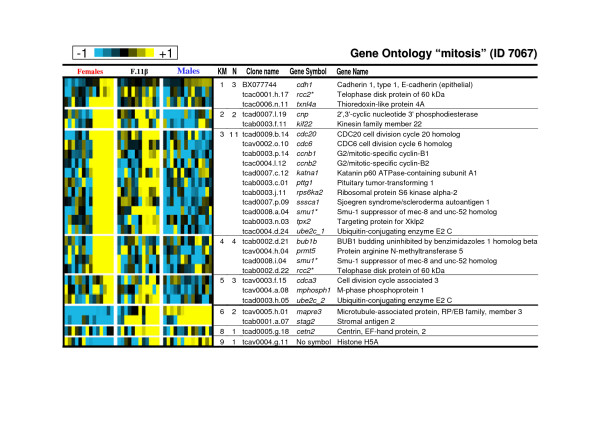
**Expression profiles of genes belonging to the Gene Ontology term "mitosis" (ID 7067)**. Clones with similar gene names that belong to the same contig (ensemble of clones with overlapping sequences) but displayed very different expression profiles were excluded from the table (when the expression profiles were not too different the gene symbol are marked with an asterisk). Clones with similar gene names and belonging to different contigs were annotated as gene symbol_1 and gene symbol_2 as they could be considered as potentially duplicated genes or differential splicing forms. KM: K-means cluster number. N: number of genes belonging to the GO term in the specified cluster.

**Figure 5:**
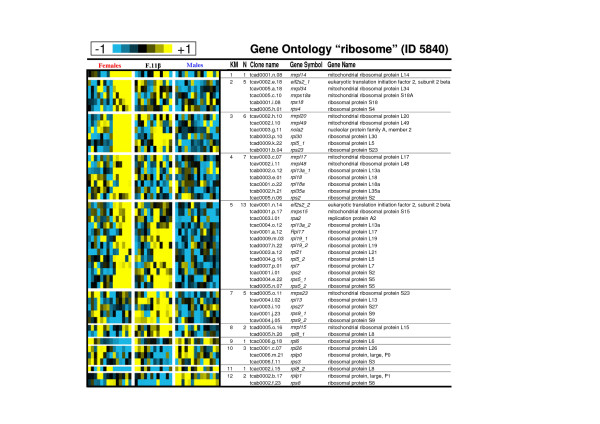
**Expression profiles of genes belonging to the Gene Ontology term "ribosome" (ID 5840)**. Clones with similar gene names that belong to the same contig (ensemble of clones with overlapping sequences) but displayed very different expression profiles were excluded from the table (when the expression profiles were not too different the gene symbol are marked with an asterisk). Clones with similar gene names and belonging to different contigs were annotated as gene symbol_1 and gene symbol_2 as they could be considered as potentially duplicated genes or differential splicing forms. KM: K-means cluster number. N: number of genes belonging to the GO term in the specified cluster.

These GO terms were selected as the 5 most significant GO terms enriched in each K-means cluster (KM) 1 to 5 with a P-value < 0.05 and a relative enrichment RE > 2 (except for the analysis of the fusion of clusters 1 to 5). n/N: number of genes assigned with a specific GO term in the cluster (n) with regards to the number of all genes assigned with the same GO term in the 2,474 analyzed genes (N). Interesting GO terms in regard to gonad differentiation and development are underlined. Genes with GO terms in bold type are detailed in figures 3 to 5. GO categories containing fewer than 5 genes were excluded.

**Table 2:** The top 5 most significant Gene Ontology (GO) terms significantly enriched in K-means clusters 6 to 13

**KM**	**Term (GO ID)**	**P-Value**	**RE**	**(n/N)**
6	cell surface (9986)	0.0000	12.30	(5/11)
	T cell activation (42110)	0.0005	16.20	(3/5)
	immune response (6955)	0.0015	4.50	(6/36)
	lipid binding (8289)	0.0020	5.20	(5/26)
	neuron development (48666)	0.0022	6.70	(4/16)
7	**extracellular matrix **(sensu Metazoa) (5578)	0.0000	4.60	(10/32)
	actin binding (3779)	0.0002	4.50	(8/26)
	muscle contraction (6936)	0.0004	6.70	(5/11)
	phosphate transport (6817)	0.0006	8.40	(4/7)
	structural molecule activity (5198)	0.0012	2.30	(15/96)
8	condensed chromosome (793)	0.0005	15.50	(3/5)
	response to endogenous stimulus (9719)	0.0025	5.00	(5/26)
	DNA repair (6281)	0.0061	5.20	(4/20)
	magnesium ion binding (287)	0.0087	4.70	(4/22)
	chromosome (5694)	0.0224	3.00	(5/43)
9	calcium ion binding (5509)	0.0004	2.50	(15/63)
	organ morphogenesis (9887)	0.0006	2.60	(13/52)
	plasma membrane (5886)	0.0011	2.10	(18/90)
	organ development (48513)	0.0018	2.10	(16/79)
	structural constituent of cytoskeleton (5200)	0.0020	4.80	(5/11)
10	urogenital system development (1655)	0.0060	7.00	(3/6)
	lipid binding (8289)	0.0080	3.20	(6/26)
	response to temperature stimulus (9266)	0.0100	6.00	(3/7)
	protein kinase binding (19901)	0.0100	6.00	(3/7)
	circulation (8015)	0.0152	5.20	(3/8)
11	metallopeptidase activity (8237)	0.0013	5.40	(5/12)
	proteolysis (6508)	0.0016	2.40	(13/69)
	peptidase activity (8233)	0.0022	2.50	(12/63)
	endopeptidase activity (4175)	0.0079	2.50	(9/47)
	transcription from RNA polymerase II promoter (6366)	0.0381	2.00	(8/51)
12	ligase activity, forming carbon-nitrogen bonds (16879)	0.0050	3.00	(7/21)
	metallopeptidase activity (8237)	0.0061	3.80	(5/12)
	mRNA metabolism (16071)	0.0165	2.10	(10/44)
	glutamine family amino acid biosynthesis (9084)	0.0200	4.60	(3/6)
	regulation of RNA metabolism (51252)	0.0322	3.90	(3/7)
13	steroid biosynthesis (6694)	0.0020	10.80	(3/9)
	sex differentiation (7548)	0.0028	9.70	(3/10)
	transcription, DNA-dependent (6351)	0.0050	2.30	(11/156)
	hormone metabolism (42445)	0.0063	7.50	(3/13)
	DNA binding (3677)	0.0071	2.20	(11/163)

**Figure 6:**
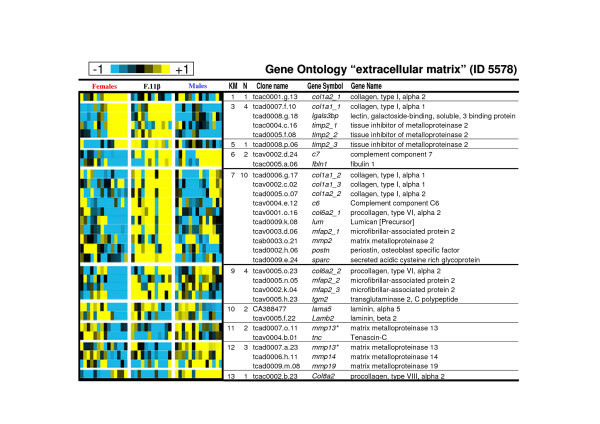
**Expression profiles of genes belonging to the Gene Ontology term "extracellular matrix" (ID 5578)**. Clones with similar gene names that belong to the same contig (ensemble of clones with overlapping sequences) but displayed very different expression profiles were excluded from the table (when the expression profiles were not too different the gene symbol are marked with an asterisk). Clones with similar gene names and belonging to different contigs were annotated as gene symbol_1 and gene symbol_2 as they could be considered as potentially duplicated genes or differential splicing forms. The 15 different contigs with homologies to zona pellucida protein homologs (zp2, zp3 and zp4 genes that are highly represented in clusters 1 to 5 have been removed from that table). KM: K-means cluster number. N: number of genes belonging to the GO term in the specified cluster.

These GO terms were selected as the 5 most significant GO terms enriched in each K-means cluster (KM) 6 to 13 with a P-value < 0.05 and a relative enrichment RE > 2. n/N: number of genes assigned with a specific GO term in the cluster (n) with regards to the number of all genes assigned with the same GO term in the 2,474 analyzed genes (N). Interesting GO terms in regard to gonad differentiation and development are underlined. Genes with GO terms in bold type are detailed in figure 6. GO categories containing fewer than 5 genes were excluded.

**Figure 7:**
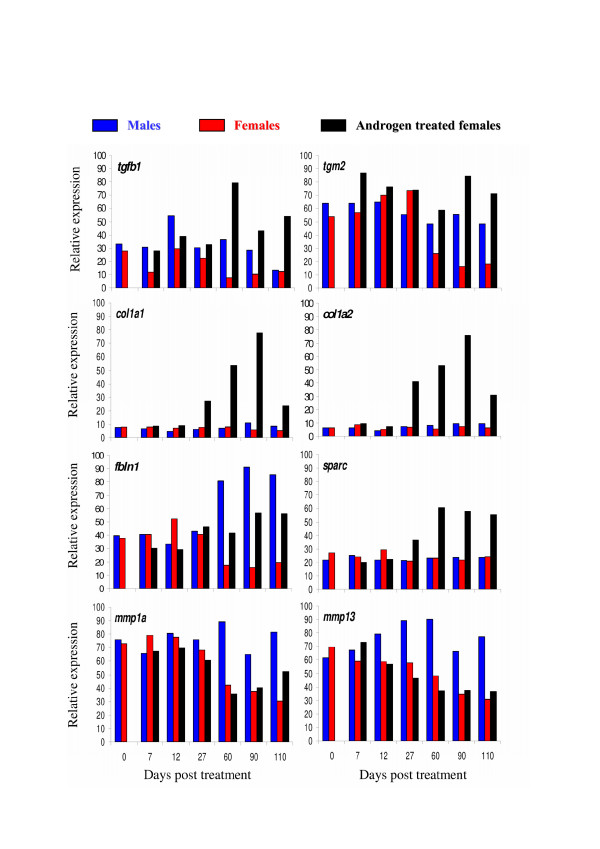
**Expression profiles of some representative genes within the GO category: extracellular matrix (ID 5578)**. Expression profile values were extracted from the DNA microarray dataset and normalized to the highest signal value observed among all samples for each gene. This highest signal value was arbitrarily set at 100 and the resulting values are designated the relative signal intensity for the studied gene at the indicated time points. Expression profiles of transforming growth factor-beta1, *tgfb1 *(AJ007836) were obtained by real-time RT-PCR. *tgm2*: transglutaminase 2, C polypeptide. *col1a1*: collagen, type I, alpha 1. *col1a2*: collagen, type I, alpha 2. *fbln1*: fibulin 1. *sparc*: secreted acidic cysteine rich glycoprotein. *mmp1a*: matrix metalloproteinase 1a. *mmp13*: matrix metalloproteinase 13.

**Figure 8:**
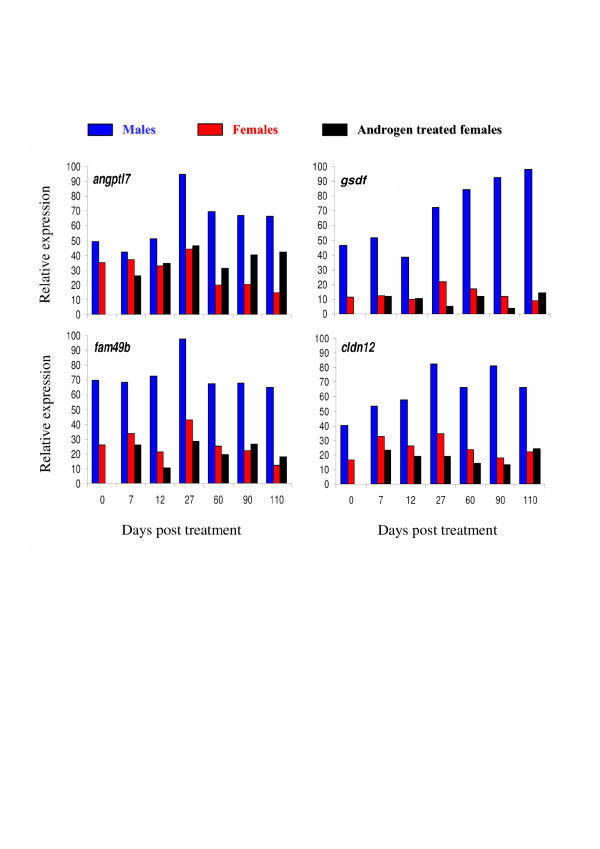
**Expression profiles of some representative genes from cluster 13**. Expression profile values were extracted from the DNA microarray dataset and normalized to the highest signal value observed among all samples for each gene. This highest signal value was arbitrarily set at 100 and the resulting values are designated the relative signal intensity for the studied gene at the indicated time points. *angiopoietin-like 7 *(*angptl7*, tcad0004.b.24 and tcac0004.n.11), *gonadal soma-derived growth factor *(*gsdf*, tcac0002.i.22), *family with sequence similarity 49, member b *(*fam49b*, tcac0003.e.03), *claudin 12 *(*cldn12*, tcad0009.h.07).

As gene expression in clusters 1 to 5 displayed similar expression profiles in the control female group, these clusters have been analysed both all together and separately (Table 1). If considered as a homogenous cluster it displays considerable enrichments in GO terms likely to be important characteristics of these late ovarian stages. Among these GO terms the most representatives (see Table 1 and Additional file 3) are the 'regulation of progression through cell cycle' (GO ID 74) [including 'mitotic cycle' (ID 278)], the 'macromolecule biosynthesis' (ID 9059) [including 'protein biosynthesis' (ID 6412)] and the cellular component ontology 'nucleolus' (ID 5730, see figure 3 for a detailed composition of this GO term). GO term 'mitosis' (ID 7067) is highly over-represented in cluster 3 (see Table 1 and figure 4 for a detailed composition of this GO term) with a 2.1 fold relative enrichment (p value of 4.10^-3^). Among clusters 1 to 5, only genes from cluster 5 are also highly expressed in the androgen-treated group. The main GO theme of cluster 5 is related to translation, with over-representation of GO 'ribosome' (ID 5840, see figure 5 for a detailed composition of this GO term), 'ribonucleoprotein complex' (ID 30529), and 'ribosome biogenesis and assembly' (ID 42254). Within the GO term 'ribosome', a large proportion of genes (7 out of 13) up-regulated in cluster 5 are also annotated as 'cytosolic large ribosomal subunit' (ID 5842, e.g. *rpl5*, *rpl7*, *rpl13a*, *rpl17*, *rpl19 *and *rpl21*, see Fig. 5). Cluster 6 contains genes that are up-regulated both in control males and androgen-treated females. It contains genes (Table 2) involved in 'cell surface' (ID 9986), 'T cell activation' (ID 42110) and the 'immune response' (ID 6955). Clusters 7 and 8 are of particular interest as they display an up-regulation only in androgen-treated females. Cluster 7 is characterized by the GO terms 'extracellular matrix' (ID 5578), 'actin binding' and 'muscle contraction' (ID 3779 and ID 6936) including genes like myosins (e.g. *myl6 *and *myl11*), tropomyosins (e.g. *tpm1*, *tpm3 *and *tpm4*) and some muscle markers (e.g. calponin2, *cnn2 *and transgelin, *tagln*). The GO term 'extracellular matrix' (Fig. 6) contains 30 different genes, and 10 are specifically up-regulated in cluster 7 following androgen treatment in females (4.6 fold enrichment with a p value < 4.10^-4^). Many of these genes (e.g. *col1a1*, *col1a2*, *col6a2*, and *mfap2*), are 'structural constituents of the extracellular matrix' (ID 5201), or are involved in 'cell adhesion' (ID 7155) (e.g. *col6a2*, *sparc *and *postn*). The expression profiles of some of these genes related to the extracellular matrix are shown in figure 7 along with the *tgfb1 *expression profile that also shows a slight up-regulation from D60 to D110 following androgen treatment in females. Cluster 8 contains genes related to 'chromosome' (ID 5694), 'condensed chromosome' (ID 793) but also, albeit only a few (2 out of 6), to the 'progression through the first phase of meiosis' ('meiosis I', ID 7126, i.e., *rad1 *and the *synaptonemal complex central element protein 2*, *syce2*). Cluster 9 is characterized by enrichment in the GO terms 'organ morphogenesis' (ID 9887), 'development' (ID 48513) and 'structural constituent of cytoskeleton' (ID 5200). Cluster 10 contains genes involved in 'urogenital system development' (ID 1655) and 'lipid binding' (ID 8289). Clusters 11 and 12 contain genes that are also assigned with the GO term 'extracellular matrix' but more specifically in relation with degradation of this extracellular matrix i.e., GO term 'metallopeptidase' (5.4 and 3.8 fold enrichment respectively) with at least 3 matrix metallopeptidases (e.g. *mmp13*, *mmp14*, *mmp19*) that are directly involved in 'collagen catabolism' (ID 30574). Cluster 13 contains genes related to 'sex differentiation' (ID 7548) (e.g. *amh*, *sox9*, *dmrt1*, *gata4*, *lhx9*), and 'steroid biosynthesis' (ID 6694) (e.g. *cyp17a1*, *cyp11b2*, *star *and *nr5a2*). According to these annotations this cluster is likely to contain other important genes also involved in testicular differentiation or in steroidogenesis regulation. Several expression profiles for such potential genes are shown in figure 8.

### Validation and enrichment of DNA microarray data by real-time RT-PCR

**Figure 9:**
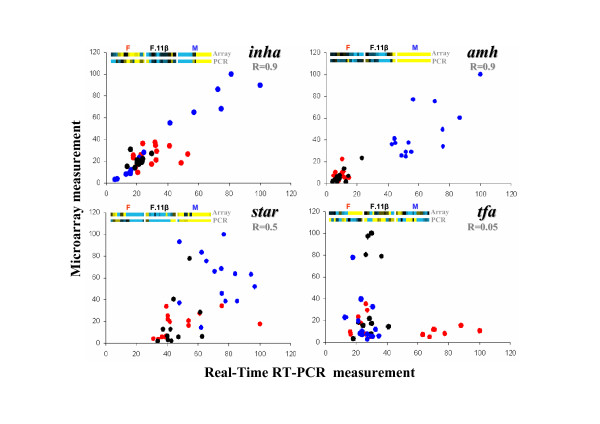
**Scatterplots of DNA microarray and real-time RT-PCR measurements for inha (inhibin alpha), amh (anti-müllerian hormone), star (Steroidogenic acute regulatory protein) and tf (transferrin)**. Each value is represented as the percentage of the highest value in each experiment (DNA microarray or real-time RT-PCR) in all experimental groups (red for females; blue for males; black for androgen-treated females). For each gene, the correlation coefficient R and colorized vectors of microarray and real-time RT-PCR values are given.

Expression profiles of 102 genes, involved in early gonad development, were measured by real-time RT-PCR. These genes all belonged to the 13 distinct k-means clusters (see Additional file 1). Among these 102 genes, 84 were only measured by real-time RT-PCR and these gene expression profiles were thus added to the microarray dataset. The remaining 18 genes were common between the real-time RT-PCR dataset and the microarray dataset (see Table 3) and were used to validate our microarray dataset. Among the 18 common genes belonging to 11 out of the 13 distinct k-means clusters (e.g. 1, 2, 3, 4, 5, 6, 8, 9, 10, 11, and 13), 15 expression profiles were found to have a significant correlation between the two techniques (see Table 3 and Fig. 9). Only *tfa *(*transferrin*), *timp2 *(*tissue inhibitor of metalloproteinase 2*) and *bzrp *(*benzodiazepine receptor, peripheral*) expression profiles did not significantly correlate. As a result, among the 13 k-means groups, at least 10 groups (e.g. 1, 2, 3, 4, 5, 6, 8, 9, 11, and 13) contained genes with significant correlation between real-time RT-PCR and DNA microarray measurements.

**Table 3:** Correlation between real-time RT-PCR and DNA microarray data

**Gene symbol**	**Gene name**	**Mouse**	**Human**	**GenBank #**	***r***	***p***	**KM**
*amh*	anti-müllerian hormone	*Amh*	AMH	BX65284			**13**
				tcav0004c.11	0.903	***	
*star*	steroidogenic acute regulatory protein	*Star*	STAR	A047032			**13**
				tcad0007a.07	0.546	***	
				tcad0008a.24	0.603	***	
*dmrt1*	doublesex- and mab-3-related transcription factor 1	*Dmrt1*	DMRT1	AF209095			**9**
				tcad0009a.11	0.813	***	
*nr5a1b*	nuclear receptor subfamily 5, group A, member 1 b	*Nr5a1*	NR5A1	AY879314			**11**
				tcad0006a.10	0.439	***	
*apoeb*	apolipoprotein E b	*Apoe*	APOE				
				AJ132620			**6**
				tcac0005c.15	0.634	***	
				tcad0007a.20	0.695	***	
*bzrp*	benzodiazepine receptor, peripheral	*Bzrp*	BZRP	AY029216			**9**
				tcab0001c.16	0.181	NS	
*bmp7*	bone morphogenetic protein 7	*Bmp7*	BMP7	BX301016			**3**
				tcav0001c.14	0.557	***	
*gdf9*	growth differentiation factor 9	*Gdf9*	GDF9	BX79301			**3**
				tcad0007a.06	0.660	***	
*inha*	inhibin alpha	*Inha*	INHA	AB044566			**6**
				tcav0001c.05	0.885	***	
				tcav0005c.12	0.907	***	
				tcac0005c.12	0.780	***	
				tcad0005a.05	0.805	***	
*gcl*	germ cell-less homolog (Drosophila)	*Gcl*	GCL	BX081118			**8**
				tcad0009a.22	0.417	***	
*vldr*	very low density lipoprotein receptor	*Vldr*	VLDR	AJ003117			**3**
				tcad0005a.13	0.823	***	
*hsp90b*	heat shock protein 90 beta	*Hspcb*	HSPCB	BX072707			**2**
				tcac0006c.15	0.628	***	
*gapdh*	glyceraldehyde-3-phosphate dehydrogenase	Gapdh	GAPDH	AF027130			**1**
				tcab0001c.21	0.427	***	
				tcac0004c.07	0.463	***	
				tcac0006c.01	0.436	***	
				tcad0002a.13	0.314	*	
*tfa*	transferrin	Trf	TF	D89083			**10**
				tcad0003a.22	0.053	NS	
*aldob*	aldolase b, fructose-bisphosphate	Aldob	ALDOB	BX081681			**3**
				tcad0001a.21	0.790	***	
*m agoh*	mago-nashi homolog, proliferation-associated	Magoh	MAGOH	BX080533			**4**
				tcab0001c.07	0.812	***	
*timp2*	tissue inhibitor of metalloproteinase 2	Timp2	TIMP2	CA360907			**9**
				tcac0004c.16	0.082	NS	
				tcad0001a.20	0.167	NS	
				tcad0005a.08	0.031	NS	
				tcad0006a.16	0.013	NS	
				tcad0007a.18	0.060	NS	
*tra2a*	transform er-2 alpha	Tra2a	TRA2A	BX074515			**5**
				tcad0008a.18	0.366	**	

Each gene is depicted by its gene symbol and gene name (according to the zebrafish nomenclature). Symbols for mouse and human homolog genes are also given with their corresponding GenBank accession number. K-means cluster number (KM) is given for each gene with the correlation coefficient r to compare correlation between expression profiles measured by real-time RT-PCR (GenBank # in bold) and microarrays (clone numbers). Significant correlations were determined using the Pearson's correlation coefficient test (**: p < 0.01; ***: p < 0.05; NS: Not Significant).

## Discussion

Our global approach based on gene expression profiling clearly reveals that, in our experimental conditions (11βOHΔ4, 10 mg/kg of food for 3 months), the androgen masculinization does not induce a natural physiological response since the transcriptome of testicular trans-differentiating gonads is quite different from the one observed during natural testicular differentiation. These differences might be due to the non physiological dosage of androgen used in our experiment. A similar study using a lower dosage may help to clarify this issue, but this study was first designed with an androgen dosage that is commonly used in rainbow trout aquaculture conditions. Whether the observed gene expression dysregulations are the reflection of a direct action of the androgens on the gonad, an indirect retro-control on the hypothalamus-pituitary axis, or a conjunction of both, remains to be elucidated. However, the synthesis of Gonadotropin Releasing Hormone (GnRH) and of pituitary hormones is established very early during rainbow trout ontogenesis [13] with at least the synthesis of Follicle Stimulating Hormone FSH [14]. Thus indirect feedback effects cannot be totally excluded.

Due to the lack of specific Gene Ontology (GO) annotation for rainbow trout we linked the best blast hits of each clone sequence with a cross-species GO annotation. This strategy relies on the accuracy of the blast homology search and also on the resulting accuracy of the GO annotations with regards to their use in a fish species. However, even if this could lead to potential errors on a gene per gene scale, the global analysis and stringent statistical screen that we carried out enabled us to unambiguously assign most clusters with a clear biological theme. Among these GO categories, some were considered as biologically informative – i.e., not too general like for instance GO "physiological process" – and robust as they contain a sufficient number of different genes to support a potential biological meaning. We focused our analysis on these biologically informative GO categories.

With regards to the effects on the gonad, our analysis first reveals that female development is highly affected by the androgen treatment, with a down-regulation of most of the genes involved in early oogenesis stages. However within this analysis we did not characterize any cluster of early female-specific up-regulated genes potentially involved in ovarian differentiation. Expression profiles of some female-specific candidate genes [15] (e.g. *foxl2a *and *foxl2b*, *cyp19a1*, *fst*, *inha*) were introduced in our analysis and all these genes were strongly and quickly inhibited by the masculinizing androgen treatment. But when pooled with our DNA microarrays dataset they did not form a tight cluster. This is probably because no additional similar expression profile was found within the DNA microarray dataset. This very small number of early female-specific genes is in agreement with the small number of candidate genes known to be involved in the ovarian differentiation pathway [16] in comparison with the relatively high number of genes that are known to characterize testicular differentiation [17]. In agreement with this view, our analysis clearly characterizes a cluster displaying testicular-specific gene expression profiles, containing both genes known to be involved in testicular differentiation (e.g. *amh*, *sox9*, *dmrt1*, *gata4*, *lhx9*) [18] and some potential new players revealed by our analysis. Interestingly the expression levels of all these genes are not restored by the androgen masculinizing treatment, and this could indicate that they are probably not necessary for early testicular differentiation in rainbow trout.

Among the gene clusters specifically up-regulated in females following masculinization with androgens, extracellular matrix, muscle markers/cytoskeleton and meiosis were characterized as the 3 main gene annotations. Simultaneous up-regulation of extracellular matrix protein genes expression and down-regulation of matrix proteinase genes was detected in gonads of androgen-treated females. At the same time the histological analysis of these gonads showed that they contain a predominant stroma of conjunctive tissue with fibroblast like cells. Matrix protein synthesis and the concomitant decrease in matrix proteinase activity have been well described as a characteristic fibrotic response of an excessive Transforming Growth Factor-beta (TGFβ) production [18,19]. Of special interest in that context is the up-regulation of transforming growth factor-β1 (*tgfb1*) in gonads of androgen-treated animals. In rat, TGFβ induced morphological changes in Leydig cells, accompanied by an increased secretion of fibronectin, laminin and collagen IV [20]. In fibroblasts treated with TGFβ1 a similar over-expression of genes associated with matrix formation has been detected including many different matrix protein genes, like SPARC (Secreted Protein, Acidic and Rich in Cysteine), MGP (matrix Gla protein), and TGFβ1 itself [21], that we also detected as up-regulated in gonads following androgen treatment. It could then be hypothesized that this late androgen up-regulation of *tgfb1 *in trout gonads triggers a fibrotic response. Surprisingly, these effects are detected transiently and rather late after the application of the androgen treatment (but concomitantly with *tgfb1 *up-regulation). Whether this reflects a total dysregulation or an exacerbation of a testicular-specific event remains to be analyzed. However, extracellular matrix deposition is known as a major event for the testicular organization. For instance, LAMA5 (Laminin α5) has been characterized as a structural protein involved in the formation of the basement membrane of the testicular cords [22] and this protein was found to be anti-correlated with Anti-Müllerian Hormone (AMH) [23]. In trout gonads, *amh *expression is not restored to male levels in androgen-treated females. This may produce a disrupted expression of some structural proteins, like *lama5*. In the same manner, *sparc *is highly up-regulated in androgen-treated females. In mouse *Sparc *gene expression has been identified in pre-Sertoli cells at the time of sex differentiation [24] and this protein has also been postulated to play a crucial role in both Leydig and Sertoli cells differentiation by affecting their morphology [25]. Structural proteins including matrix proteins are then of major importance for a complete and functional testicular differentiation and their up-regulation in trout following an androgen treatment inducing testicular transdifferentiation may be the consequence of a dysregulation of some major regulators of their synthesis like *amh *or *tgfb1*.

We also detected a high number of genes associated with cytoskeletal reorganization and muscle development that were up-regulated by the treatment. Some of them (e.g. *cnn1*, *myh11*, *myl6*, *tagln*) are even considered as characteristic smooth muscle markers. This expression of muscle markers in the testis is likely in relation with the peritubular myoid cells that surround the seminiferous tubules [26]. These myoid cells are known to express muscle markers like for instance, *tpm1 *(*tropomyosin 1, alpha*) [27], smooth muscle alpha-actin [28], and smooth muscle myosin [29]. Differentiation of these cells is androgen dependent [28] and they contribute to the testicular secretion of extracellular matrix components [30] along with the Sertoli cells [31]. It is therefore suggested that the masculinizing androgen treatment may induce the differentiation and subsequently a disturbed androgen-dependent proliferation of these peritubular myoid cells. These cells are also probably involved in the important extracellular matrix synthesis that occurs concomitantly with this differentiation.

In our experiment, the androgen treatment also induced a precocious spermatogenesis as revealed both by the histological analysis and by the increased expression levels of some genes involved in testicular meiosis. In fish, androgens and particularly, 11-oxygenated androgens, are strongly involved in spermatogenesis regulation [32] and they have been shown to directly induce spermatogenesis *in vitro *in some species [33]. Similarly, in mammals, three independent studies using Sertoli cell-specific AR-knockout mice (mice knockout for the androgen receptor, AR) demonstrated that the action of androgen is an absolute requirement for the completion of spermatogenesis, particularly in the process of meiosis [34-36].

## Conclusion

This study gives a first comprehensive survey of gene expression during androgen-induced masculinization in female rainbow trout. Our data provide supportive evidences that this treatment results in a marked dysregulation of gene expression levels when compared to natural testicular or ovarian differentiation. In our experimental condition the androgen treatment induces the complete down-regulation of female specific genes, but not the complete restoration of the male-specific gene expression patterns. Instead, some disturbed responses were characterized by an exacerbation of extracellular matrix synthesis and muscle type cell differentiation and proliferation (myoid cells) followed by a precocious meiosis of germ cells. All together, we suggest that androgen masculinization acts mainly through an early inhibition of female development rather than through a direct induction of testicular differentiation.

## Methods

### Animals and samplings

Research involving animal experimentation has been approved by the authors' institution (authorization no. 35-14). It conforms to principles for the use and care of laboratory animals and is in compliance with French and European regulations on animal welfare (European Convention for the Protection of Vertebrate Animals Used for Experimental and Other Scientific Purposes, ETS no. 123, January 1991). All-male and all-female rainbow trout populations were obtained at the INRA experimental fish farm (Sizun, France) as previously described [37]. Treatment with androgens (female treated Group 'F11β') was carried out at the onset of the first feeding [Day 0 = D0 at 55 days post-fertilization (55 dpf)], on an all-female population. The androgen, 11β-hydroxyandrostenedione (11βOHΔ4, Sigma, St. Louis, MO, USA), was administered by adding it to the food (10 mg/kg food) during 3 months starting from the first feeding and this treatment has been shown to produce 100% sex-inversions [10]. In each group, 20 to 100 gonads were sampled and pooled in duplicates corresponding to the various stages of development: onset of the free swimming period after complete yolk resumption (Day 0 = D0), D0+7 days (D7), occurrence of oocyte meiosis (D12), beginning of ovarian lamellar structures development (D27), occurrence of previtellogenic oocytes (D60), D90 and D110. They were immediately frozen in liquid nitrogen and stored at -80°C until RNA extraction. Additional gonads were sampled at the same time-points for histological analysis, which was performed as previously described [38].

### Total RNA extraction

Total RNA was extracted using TRIzol reagent (Invitrogen, Cergy Pontoise, France) as previously described [39]. The total RNA concentration was determined with an Agilent 2100 Bioanalyzer and the RNA 6000 LabChip^® ^kit (Agilent Technologies, Stockport, UK) according to the manufacturers' instructions.

### DNA microarrays preparation

#### DNA Microarrays construction

Gene expression analyses were carried out using home-made Nylon DNA microarrays using a previously described technology [40]. These DNA microarrays were built using as templates cDNA clones provided by the INRA-AGENAE program [7]. All these clones were PCR-amplified at the INRA Resources Centre for Animal Genomics (CRB GADIE, Jouy en Josas, France) using primers designed on the plasmid sequences flanking the cDNA inserts (M13RP1 5'-GTGGAATTGTGAGCGGATAAC and M13RP2 5'-GCAAGGCGATTAAGTTGGG). 35 cycles of PCR amplifications were carried out in 100 μl of 1× buffer containing 25 mM MgCl2, 250 μM dNTP, 100 μM of each primer, and 2.5 Units of Taq polymerase (Promega, Madison, WI). For each cDNA clone, two 100 μl PCR reactions were pooled, desiccated and resuspended in 50 μl of distilled water. They were then spotted as previously described [41] onto Hybond-N+ 2 × 7 cm^2 ^membranes (Amersham Pharmacia Biotech, Cleveland, OH, USA) attached to glass slides using an 8-pin print head (Pin-and-Ring™ technology) on the GMS 417™ (Affymetrix, MWG-Biotech, Ebersberg, Germany). Spotted DNA was then denatured and UV – cross-linked onto nylon filters. All DNA microarrays used in this study were made at the same time and under the same conditions. These trout microarrays contained 9,216 DNA spots representing 9,120 trout cDNA clones and a set of 96 controls. Among these cDNA clones, 7,584 were issued from a pooled-tissues library and 1,536 from a testis library [7]. Negative controls consisted of 80 spots of an *Arabidopsis thaliana *cytochrome c554 clone which is devoid of similarity with trout DNA sequences, 8 spots of poly(dA)80 and 8 spots of PCR reaction without template.

#### DNA microarray hybridizations

Microarrays were hybridized with two types of 33P-labeled probes. The first one was an oligonucleotide with a sequence common to all spotted PCR-products (vector hybridization) in order to determine the amount of target DNA accessible to hybridization in each spot. After stripping, a second hybridization was performed with complex probes made from 1 μg of retrotranscribed total RNA [40-42]. Protocols for probes preparation, hybridizations and washes are available online [43]. After stringent washes, arrays were exposed to phosphor-imaging plates and scanned with a FUJI BAS 5000 at 25 μm resolution. Hybridization signals were quantified using ArrayGauge software (Fuji Ltd, Tokyo, Japan).

### Real-time RT-PCR

In order to validate and enrich the DNA microarray dataset, expression of 102 genes involved in early gonad development [15] was measured by real-time reverse transcription-polymerase chain reaction (RT-PCR). For cDNA synthesis, 1 μg of total RNA was denatured in the presence of random hexamers (0.5 μg) for 5 min at 70°C, and then chilled on ice. Reverse transcription (RT) was performed at 37°C for 1 h using M-MLV reverse transcriptase (Promega, Madison, WI, USA) as described by the manufacturer. Real-time PCR was carried out as previously described [15] using the iCycler iQTM (Bio-Rad, Hercules, CA, USA) and the SYBER Green PCR master Mix (Eurogentec, Seraing, Belgium). For each target gene, all the samples were analyzed on the same plate in the same PCR assay. PCR data were processed as previously described, each transcript level being normalized by division with the expression values of the constitutive elongation factor 1α (*ef1a*), which was used as an internal standard [15]. Data were then included in the microarray data matrix for clustering analysis (see next paragraph).

### Data analysis

First, non-linear effects such as background, print-tip effects or saturation were corrected by LOWESS [44], using a channel by channel procedure [45]. Each array was individually normalized to the median profile of all arrays. We used the print-tip LOWESS version implemented in the statistical software package R [46]. Data were further corrected for the amount of spotted cDNA. This step is necessary as it has been shown that the signal intensity is proportional to the amount of probe on the surface of the array [40,47]. This effect can be observed both for glass and Nylon surfaces. This effect is corrected by the use of a reference in dual channel arrays, and by an independent measurement of the spotted amount of DNA probe in single channel arrays. On Nylon membranes, this effect is linear and can be corrected by dividing the signal by the amount of probe [40]. Briefly, sample signal intensity of each spot ("S") was divided ("S/V") by the corresponding signal intensity of the same spot obtained with the vector hybridization ("V"). To minimize experimental differences between different complex probe hybridizations, 'S/V' values from each hybridization were divided by the corresponding median value of 'S/V' (quantile normalization).

A triple filtering procedure was then applied to the microarray dataset. The first consisted of filtering background signals due to low amount of spotted DNA. When a "V" spot signal was too weak (vector signal < 3× vector local background), the data of the corresponding cDNA clone was discarded (missing data). The second filtering procedure was applied to eliminate non informative genes that were not measured (sample signal < 3× sample local background) in more than 20% of the samples. Finally, genes exhibiting little variation (coefficient of variation < 0.1) across all arrays were excluded from the analysis [48,49]. After these three filtering steps, 2,372 genes were retained for further analysis.

All data (2372 microarray and 102 real-time RT-PCR gene expression profiles) were then log2-transformed and were analyzed by unsupervised and supervised clustering methods. Hierarchical clustering (Cluster program [50]) investigated the relationships between the genes and between the samples by using centroid linkage clustering with Pearson's uncentered correlation as similarity metric on data that were median-centered on genes. Gene clusters were distinguished using the non-hierarchical unsupervised learning k-means algorithm implemented in the Cluster program [50]. It was run on log2-transformed and gene median-centered data with a maximum cycles parameter of 100. The optimal minimal 'k' number of clusters, corresponding to the stability of the k-means clustering, was empirically set at 13. Indeed, with smaller k numbers, some clusters merged together whereas with greater k numbers, the size of some clusters decreased (less than 50 genes to truly empty clusters). Results (colorized matrix) of hierarchical and k-means clustering analyses were visualized using the Java TreeView program [51]. Functional annotation of genes was performed using Gene Ontology [52] and the GoMiner program [53]. Significance of over- or under-representation was calculated using Fisher's exact test at 0.05% risk.

## Competing interests

The author(s) declares that there are no competing interests.

## Authors' contributions

DB and JM designed and spotted this rainbow trout microarray. DB carried out the microarray experiments and analysis with substantial help from RH for the design of the experiment and the analysis. YG and AF conceived the study, participated in its design and coordination. YG and DB drafted the manuscript. All authors read and approved the final manuscript.

## Supplementary Material

Additional file 1 **Median expression profiles of the 2,474 genes differentially expressed in androgen-treated masculinized rainbow trout females**. Median expression profiles of the 2,474 genes that display significant changes between male, female and female treated with an androgen masculinizing treatment. This dataset has been subjected to normalization (see material and methods). The K-means cluster (KM) is given for all clones with their clone name, corresponding gene annotation and their median expression levels in females, females treated with androgen and males from D0 to D110. Duplicates samples are labelled -1 and -2. Click here for file

Additional file 2 **Clone names and annotations of the 2,474 genes differentially expressed in androgen-treated masculinized rainbow trout females**. Annotation of 2,474 genes differentially expressed in females, males and androgen-treated masculinized rainbow trout females. Information provided for each clones (or for each accession number of the sequence if the data has been obtained by RT-PCR): the corresponding contig (that can be found at the following website ), the best blast hit description along with the corresponding database (Prodom or Swissprot), the score, the e-value and the percentage of identity. Click here for file

Additional file 3 **Complete Gene Ontology (GO) analysis with significantly enriched GO in clusters of correlated expression**. Significant (P-value < 0.05) GO terms and their definition for all K-means clusters (KM 1 to 13). N KM: number of genes within the corresponding KM cluster. Total: total number of genes within the selected GO term. P-Value: p-value of the significant enrichment. RE: relative enrichment. GO ID: gene ontology identification number. Term: GO terms. Click here for file

## References

[B1] Guiguen Y (2000). Implication of steroids in fish gonadal sex differentiation and sex inversion. Current Topics in Steroid Research.

[B2] Hayes TB (1998). Sex determination and primary sex differentiation in amphibians: Genetic and developmental mechanisms. Journal of Experimental Zoology.

[B3] Pieau C, Dorizzi M (2004). Oestrogens and temperature-dependent sex determination in reptiles: all is in the gonads. Journal of Endocrinology.

[B4] Smith CA, Sinclair AH (2004). Sex determination: insights from the chicken. Bioessays.

[B5] Pandian TJ, Sheela SG (1995). Hormonal induction of sex reversal in fish. Aquaculture.

[B6] Chevassus B, Devaux A, Chourrout D, Jalabert B (1988). Production of YY rainbow trout males by self-fertilization of induced hermaphrodites. Journal of Heredity.

[B7] Govoroun M, Le Gac F, Guiguen Y (2006). Generation of a large scale repertoire of Expressed Sequence Tags (ESTs) from normalised rainbow trout cDNA libraries. BMC Genomics.

[B8] Rexroad CE, Lee Y, Keele JW, Karamycheva S, Brown G, Koop B, Gahr SA, Palti Y, Quackenbush J (2003). Sequence analysis of a rainbow trout cDNA library and creation of a gene index. Cytogenetic and Genome Research.

[B9] Liu S, Govoroun M, D'Cotta H, Ricordel MJ, Lareyre JJ, McMeel OM, Smith T, Nagahama Y, Guiguen Y (2000). Expression of cytochrome P450(11beta) (11beta-hydroxylase) gene during gonadal sex differentiation and spermatogenesis in rainbow trout, *Oncorhynchus mykiss*. J Steroid Biochem Mol Biol.

[B10] Govoroun M, McMeel OM, D'Cotta H, Ricordel MJ, Smith T, Fostier A, Guiguen Y (2001). Steroid enzyme gene expressions during natural and androgen-induced gonadal differentiation in the rainbow trout, *Oncorhynchus mykiss*. J Exp Zool.

[B11] Gene Expression Omnibus database. http://www.ncbi.nih.gov/geo/.

[B12] Browseable file containing the k-means clustering. http://www.sigenae.org/fileadmin/_temp_/TreeView/troutApplet.html.

[B13] Feist G, Schreck CB (1996). Brain-pituitary-gonadal axis during early development and sexual differentiation in the rainbow trout, *Oncorhynchus mykiss*. General and Comparative Endocrinology.

[B14] Saga T, Oota Y, Nozaki M, Swanson P (1993). Salmonid pituitary gonadotrophs. III. Chronological appearance of GTH I and other adenohypophysial hormones in the pituitary of the developing trout (*Oncorhynchus mykiss irideus*). General and Comparative Endocrinology.

[B15] Baron D, Houlgatte R, Fostier A, Guiguen Y (2005). Large-scale temporal gene expression profiling during gonadal differentiation and early gametogenesis in rainbow trout. Biol Reprod.

[B16] Yao HHC (2005). The pathway to femaleness: current knowledge on embryonic development of the ovary. Molecular and Cellular Endocrinology.

[B17] Brennan J, Capel B (2004). One tissue, two fates: molecular genetic events that underlie testis versus ovary development. Nat Rev Genet.

[B18] Branton MH, Kopp JB (1999). TGF-beta and fibrosis. Microbes Infect.

[B19] Leask A, Abraham DJ (2004). TGF-beta signaling and the fibrotic response. FASEB J.

[B20] Dickson C, Webster DR, Johnson H, Millena AC, Khan SA (2002). Transforming growth factor-β effects on morphology of immature rat Leydig cells. Molecular and Cellular Endocrinology.

[B21] Chambers RC, Leoni P, Kaminski N, Laurent GJ, Heller RA (2003). Global expression profiling of fibroblast responses to transforming growth factor-beta1 reveals the induction of inhibitor of differentiation-1 and provides evidence of smooth muscle cell phenotypic switching. Am J Pathol.

[B22] Pelliniemi LJ, Fröjdman K (2001). Structural and Regulatory macromolecules in sex differentiation of gonads. Journal of Experimental Zoology.

[B23] Fröjdman K, Pelliniemi LJ, Rey R, Virtanen I (1999). Presence of anti-Müllerian hormone correlates with absence of laminin α5 chain in differentiating rat testis and ovary. Histochem Cell Biol.

[B24] Boyer A, Lussier JG, Sinclair AH, McClive PJ, Silversides DW (2004). Pre-sertoli specific gene expression profiling reveals differential expression of Ppt1 and Brd3 genes within the mouse genital ridge at the time of sex determination. Biol Reprod.

[B25] Vernon RB, Sage H (1989). The calcium-binding protein SPARC is secreted by Leydig and Sertoli cells of the adult mouse testis. Biol Reprod.

[B26] Cauty C, Loir M (1995). The interstitial cells of the trout testis (Oncorhynchus mykiss): ultrastructural characterization and changes throughout the reproductive cycle. Tissue and Cell.

[B27] Jeanes A, Wilhelm D, Wilson MJ, Bowles J, McClive PJ, Sinclair AH, Koopman P (2005). Evaluation of candidate markers for the peritubular myoid cell lineage in the developing mouse testis. Reproduction.

[B28] Schlatt S, Weinbauer GF, Arslan M, Nieschlag E (1993). Appearance of alpha-smooth muscle actin in peritubular cells of monkey testes is induced by androgens, modulated by follicle-stimulating hormone, and maintained after hormonal withdrawal. J Androl.

[B29] Paranko J, Pelliniemi LJ (1992). Differentiation of smooth muscle cells in the fetal rat testis and ovary: localization of alkaline phosphatase, smooth muscle myosin, F-actin, and desmin. Cell Tissue Res.

[B30] Maekawa M, Kamimura K, Nagano T (1996). Peritubular myoid cells in the testis: their structure and function. Arch Histol Cytol.

[B31] Raychoudhury SS, Irving MG, Thompson EW, Blackshaw AW (1992). Collagen biosynthesis in cultured rat testicular Sertoli and peritubular myoid cells. Life Sci.

[B32] Nagahama Y (1994). Endocrine regulation of gametogenesis in fish. International Journal of Developmental Biology.

[B33] Miura T, Yamauchi K, Takahashi H, Nagahama Y (1991). Hormonal induction of all stages of spermatogenesis in vitro in the male japanese eel (*Anguilla japonica*). PNAS.

[B34] Chang CS, Chen YT, Yeh SD, Xu QQ, Wang RS, Guillou F, Lardy H, Yeh SY (2004). Infertility with defective spermatogenesis and hypotestosteronemia in male mice lacking the androgen receptor in Sertoli cells. PNAS.

[B35] De Gendt K, Swinnen JV, Saunders PTK, Schoonjans L, Dewerchin M, Devos A, Tan K, Atanassova N, Claessens F, Lecureuil C, Heyns W, Carmeliet P, Guillou F, Sharpe RM, Verhoeven G (2004). A Sertoli cell-selective knockout of the androgen receptor causes spermatogenic arrest in meiosis. PNAS.

[B36] Holdcraft RW, Braun RE (2004). Androgen receptor function is required in Sertoli cells for the terminal differentiation of haploid spermatids. Development.

[B37] Guiguen Y, Baroiller JF, Ricordel MJ, Iseki K, McMeel OM, Martin SAM, Fostier A (1999). Involvement of estrogens in the process of sex differentiation in two fish species: the rainbow trout (*Oncorhynchus mykiss*) and a tilapia (*Oreochromis niloticus*). Molecular Reproduction and Development.

[B38] Baron D, Cocquet J, Xia X, Fellous M, Guiguen Y, Veitia RA (2004). An evolutionary and functional outlook of *FoxL2 *in rainbow trout gonad differentiation. Journal of Molecular Endocrinology.

[B39] Govoroun M, McMeel OM, Mecherouki H, Smith TJ, Guiguen Y (2001). 17beta-estradiol treatment decreases steroidogenic enzyme messenger ribonucleic acid levels in the rainbow trout testis. Endocrinology.

[B40] Bertucci F, Bernard K, Loriod B, Chang YC, Granjeaud S, Birnbaum D, Nguyen C, Peck K, Jordan BR (1999). Sensitivity issues in DNA array-based expression measurements and performance of nylon microarrays for small samples. Hum Mol Genet.

[B41] Bertucci F, Van Hulst S, Bernard K, Loriod B, Granjeaud S, Tagett R, Starkey M, Nguyen C, Jordan B, Birnbaum D (1999). Expression scanning of an array of growth control genes in human tumor cell lines. Oncogene.

[B42] Bertucci F, Nasser V, Granjeaud S, Eisinger F, Adelaide J, Tagett R, Loriod A, Giaconia A, Benziane A, Devilard E, Jacquemier J, Viens P, Nguyen C, Birnbaum D, Houlgatte R (2002). Gene expression profiles of poor-prognosis primary breast cancer correlate with survival. Hum Mol Genet.

[B43] Protocols for membrane microarrays. http://tagc.univ-mrs.fr/oncogenomics/Nylon_microarrays.php.

[B44] Yang YH, Dudoit S, Luu P, Lin DM, Peng V, Ngai J, Speed TP (2002). Normalization for cDNA microarray data: a robust composite method addressing single and multiple slide systematic variation. Nucleic Acids Res.

[B45] Workman C, Jensen LJ, Jarmer H, Berka R, Gautier L, Nielser HB, Saxild HH, Nielsen C, Brunak S, Knudsen S (2002). A new non-linear normalization method for reducing variability in DNA microarray experiments. Genome Biol.

[B46] Ihaka R, Gentleman R (1996). A language for data analysis and graphics. J Comput Graph Statist.

[B47] Stillman BA, Tonkinson JL (2001). Expression microarray hybridization kinetics depend on length of the immobilized DNA but are independent of immobilization substrate. Anal Biochem.

[B48] Perou CM, Sorlie T, Eisen MB, van de Rijn M, Jeffrey SS, Rees CA, Pollack JR, Ross DT, Johnsen H, Akslen LA, Fluge O, Pergamenschikov A, Williams C, Zhu SX, Lonning PE, Borresen-Dale AL, Brown PO, Botstein D (2000). Molecular portraits of human breast tumours. Nature.

[B49] Bertucci F, Salas S, Eysteries S, Nasser V, Finetti P, Ginestier C, Charafe-Jauffret E, Loriod B, Bachelart L, Montfort J, Victorero G, Viret F, Ollendorff V, Fert V, Giovaninni M, Delpero JR, Nguyen C, Viens P, Monges G, Birnbaum D, Houlgatte R (2004). Gene expression profiling of colon cancer by DNA microarrays and correlation with histoclinical parameters. Oncogene.

[B50] Eisen MB, Spellman PT, Brown PO, Botstein D (1998). Cluster analysis and display of genome-wide expression patterns. PNAS.

[B51] Saldanha AJ (2004). Java Treeview – extensible visualization of microarray data. Bioinformatics.

[B52] Ashburner M, Ball CA, Blake JA, Botstein D, Butler H, Cherry JM, Davis AP, Dolinski K, Dwight SS, Eppig JT, Harris MA, Hill DP, Issel-Tarver L, Kasarskis A, Lewis S, Matese JC, Richardson JE, Ringwald M, Rubin GM, Sherlock G (2000). Gene ontology: tool for the unification of biology. The Gene Ontology Consortium. Nat Genet.

[B53] Zeeberg BR, Feng W, Wang G, Wang MD, Fojo AT, Sunshine M, Narasimhan S, Kane DW, Reinhold WC, Lababidi S, Bussey KJ, Riss J, Barrett JC, Weinstein JN (2003). GoMiner: a resource for biological interpretation of genomic and proteomic data. Genome Biology.

